# Case Study: Safety Assessment of Plant Layout between Ethylene Storage Tanks and Process Equipment According to Capacity and Weather Conditions

**DOI:** 10.3390/ijerph17082849

**Published:** 2020-04-21

**Authors:** Jae-Young Choi, Sang-Hoon Byeon

**Affiliations:** Department of Health & Safety Convergence Science, Korea University, Anam-ro 145, Seongbuk-gu, Seoul 02841, Korea; jaeyoungchoi@korea.ac.kr

**Keywords:** safety distance, QRA, weather variable, ethylene, thermal radiation

## Abstract

In a chemical plant, even if an explosion occurs in a storage tank that handles flammable materials, the minimum separation distance is applied in a way to prevent chain explosion. This is because when an explosion occurs in a storage tank, thermal radiation affects nearby process equipment and causes a chain explosion. The separation distance between storage tanks and process equipment in a chemical plant depends on the global engineering guidelines PIP (Process Industry Practice) PNE00003 and GAPS (Global Asset Protection Services) GAP.2.5.2. However, there is a limitation in the global engineering guidelines that provide only a consistent separation distance according to the item types without considering the storage capacity and climatic conditions around the storage tank. This study analyzed the distance of thermal radiation (up to 37.5 kW/m^2^) according to the capacity of ethylene storage tank and climatic conditions by utilizing the Phast (DNV GL), which is widely used as a tool for quantitative risk analysis. The accident scenario was applied using the U.S. EPA (Environment Protection Agency)’s worst leakage accident scenario, considering climate variables, air temperature, wind speed, and atmospheric stability. In the simulation, atmospheric stability did not affect the radius of thermal radiation. Moreover, the thermal radiation tended to increase with increase in wind speed in the ambient condition and winter, but not in summer. Because both PIP PNE00003 and GAP.2.5.2 yield consistent separation distances without considering storage capacity and weather conditions, quantitative risk analysis of the results of thermal radiation is necessary to ensure safety.

## 1. Introduction

Ethylene is a representative chemical raw material used in polyethylene, ethylene glycol, PVC, etc. and can be considered the most basic material in the petrochemical industry manufactured at an NCC (Naphtha Cracking Center) or ECC (Ethane Cracking Center) [1]. When constructing a chemical plant for ethylene-related processes, the location of the plant facility must be determined by conducting a safety-distance review via a plot plan. In particular, the location of the storage tank, which handles a large amount of ethylene should be the most important when conducting a safety-distance review [2].

The locations of facilities included in the plot plan of a chemical plant are set at an early stage of designing and must be carefully reviewed because they can significantly affect the economy (the quantity of pipes and land use) [3]. The review stage is the most important stage in terms of safety distance. Because chemical plants store and handle large amounts of flammable materials, there is a risk of explosion. Therefore, the separation distance must be set by considering the characteristics of each facility to minimize the damage in the event of an explosion [4]. The safety distance is considered, in collaboration with process safety engineers, piping engineers, and engineers from other disciplines [5]. The widely used engineering guidelines related to safety distances are ‘PIP PNE00003: Process Unit and Offsites Layout Guide’ and ‘GAP.2.5.2: Oil and Chemical Plant Layout and Spacing’ [6]. PIP PNE 00003 is a standard for separation distances issued by an association called Process Industry Practice (PIP). PIP is composed of about 98 major engineering companies and provides engineering guideline by their consultations. GAP.2.5.2 is a standard for separation distances issued by an association called Global Asset Protection Services (GAPS). GAPS provides engineering guidelines from the insurance company’s point of view to prevent property loss for the owners of a chemical plants. They suggest safety distances of 46 and 106.75 m, respectively, between the ethylene storage tank and process equipment. However, there is a limitation in that both guidelines suggest a consistent separation distance without considering the capacity of ethylene storage and atmospheric conditions.

Therefore, this study aims to suggest the assessment of safety distance between ethylene storage tanks and process equipment considering the storage capacity and atmospheric conditions. In the previous study, when applying the concept of separation distance, it was suggested that the effect of thermal radiation should be considered [7]. It was noted that the effect of the thermal radiation could be analyzed by the Quantitative Risk Analysis (QRA) technique. This study also considered the effect of thermal radiation on the separation distance. As a simulation tool for the QRA technique, Phast (DNV GL) was used. Phast is widely used as a QRA simulation tool [8]. This is because Phast is designed to comply with legal requirements from around the world, such as the Dutch’s yellow book, the U.S. EPA (Environment Protection Agency) regulation, and the UK’s HSE (Health and Safety Executive) regulation [9,10,11].

## 2. Background

Ethylene is a petrochemical product that is at the center of supply and demand in the global petrochemical market. Ethylene is used in the gas phase at ambient temperature but liquefied under the conditions of low temperature and high pressure during storage and transportation [12]. This study aims to analyze the safety distance between ethylene storage tanks and process equipment. Ethylene storage tanks form a liquid pool during leakage, and in the event of a fire, the vicinity is affected by thermal radiation [13]. Globally, the effect of 37.5 kW/m^2^ of thermal radiation on process-plant facilities is considered fatal, as summarized in Table 1 [14].

Many studies have been carried out that have selected 37.5 kW/m^2^ as the criterion for the influence of thermal radiation on process equipment. There was a study that found 37.5 kW/m^2^ is suitable as a thermal radiation criterion causing damage to facilities in an experiment that applied thermal radiation to a sample of an offshore platform, and propane was applied for the source material of thermal radiation [15]. Another study selected 37.5 kW/m^2^ as the criterion for causing chain explosion under the influence of thermal radiation of process equipment and Liquefied Petroleum Gas (LPG) was applied for the source material of thermal radiation [16]. There was also a study that the separation distance between the propylene process equipment and the other facilities of chemical plant should be 37.5 kW/m^2^ of thermal radiation, and propylene was applied for the source material of thermal radiation [17]. Another study applied 37.5 kW/m^2^, the criterion of thermal radiation, which has a fatal effect on structures when an explosion occurs due to a leak in an Liquefied Natural Gas (LNG) tank, and LNG was applied for the source material of thermal radiation [18]. Therefore, this study analyzed thermal radiation up to 37.5 kW/m^2^, considering the ethylene storage capacity and atmospheric conditions. QRA is widely used for such thermal radiations [19]. This study used Phast as a QRA simulation tool. The catastrophic rupture scenario provided by Phast was considered as the leakage scenario of the ethylene storage tank [20]. In addition, EPA’s worst-case leak scenario was applied for other conditions required for the leakage scenario [21] to conservatively approach the effects of thermal radiation due to the ethylene storage tank.

## 3. Methodology and Results

**Figure 1:**
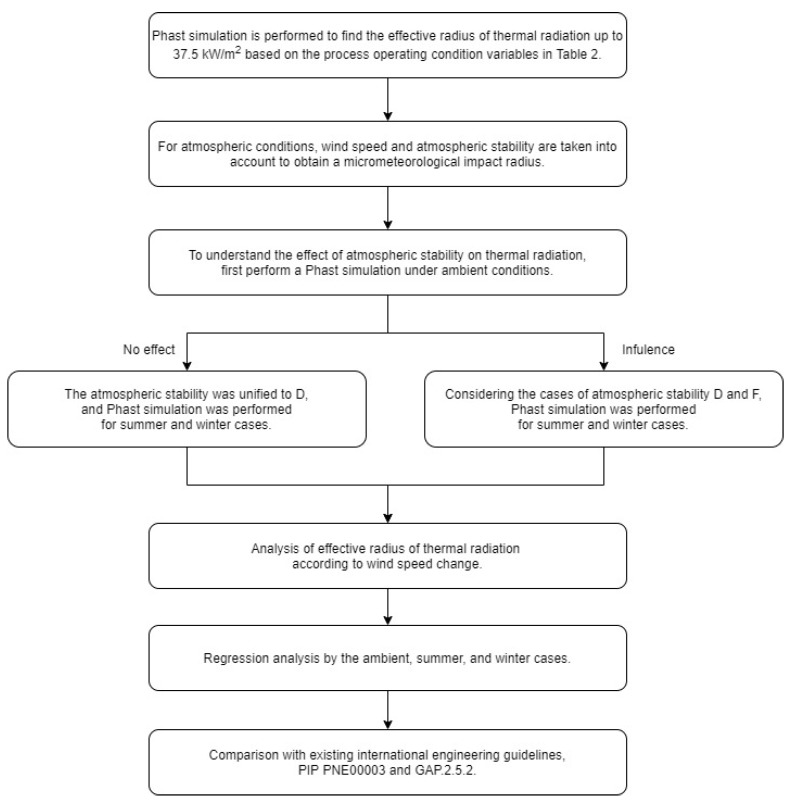
Methodology flowchart in this study.

The radius of thermal radiation (up to 37.5 kW/m^2^) was considered. The methodology flow chart applied in this study is shown in Figure 1. In this study, thermal radiation was analyzed by Phast’s Gaussian model. The Gaussian model is a representative method that is considered for the release of materials that are lighter than air. Since the source material of this study, ethylene, is lighter than air, the Gaussian model was applied. Atmospheric stability was estimated by the temperature difference according to the height and classified into seven categories from A to G. This study was applied based on atmospheric stability D and F according to EPA’s worst-case scenario.

Ethylene was applied as the target material, and the process conditions of the tank (source item) for storing ethylene in the liquid phase are presented in Table 2. Nine storage tank capacities were set from 10 to 50 m^3^ with an interval of 5 m^3^.

The height of the source of leakage in the ethylene storage tank was based on the condition of leakage from the ground and 50% relative humidity in the air according to EPA’s worst-case scenario [21]. The following atmospheric temperatures were applied—ambient temperature (25 °C), summer (40 °C), and winter (0 °C). The applied wind speed and atmospheric stability are presented in Table 3.

**Figure 2:**
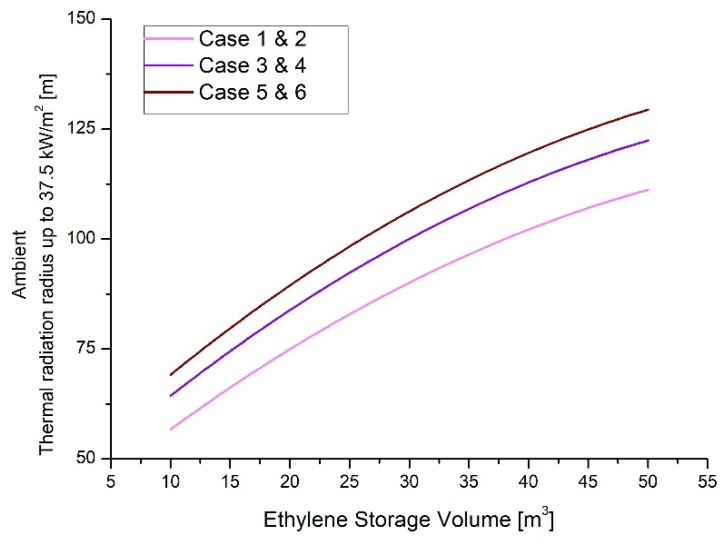
Thermal radiation radius up to 37.5 kW/m^2^ according to ethylene storage capacity at ambient temperature (25 °C).

The simulation results at ambient temperature (25 °C) are presented in Table 4 and Figure 2, and the results of regression analysis with polynomial fitting are presented in Table 5.

Regression equations for each case are expressed as Equations (1)–(3). The calculation of each equation provided results with high interpretation reliabilities.

(1) $$\mathrm{Case}\;1\;\mathrm{and}\;2\text{: }Y=35.46308+2.2785X-0.01528X^{2}$$

(2) $$\mathrm{Case}\;2\;\mathrm{and}\;3\text{: }Y=41.49652+2.44807X-0.0166X^{2}$$

(3) $$\mathrm{Case}\;4\;\mathrm{and}\;5\text{: }Y=45.27472+2.55867X-0.01752X^{2}$$

The difference in atmospheric F also did not affect the radius of thermal radiation (up to 37.5 kW/m^2^). This was true not only at ambient temperature (25 °C) but also under summer (40 °C) and winter (0 °C) conditions when the atmospheric stability was fixed at D, and simulation was performed using the atmospheric conditions in Table 6.

**Figure 3:**
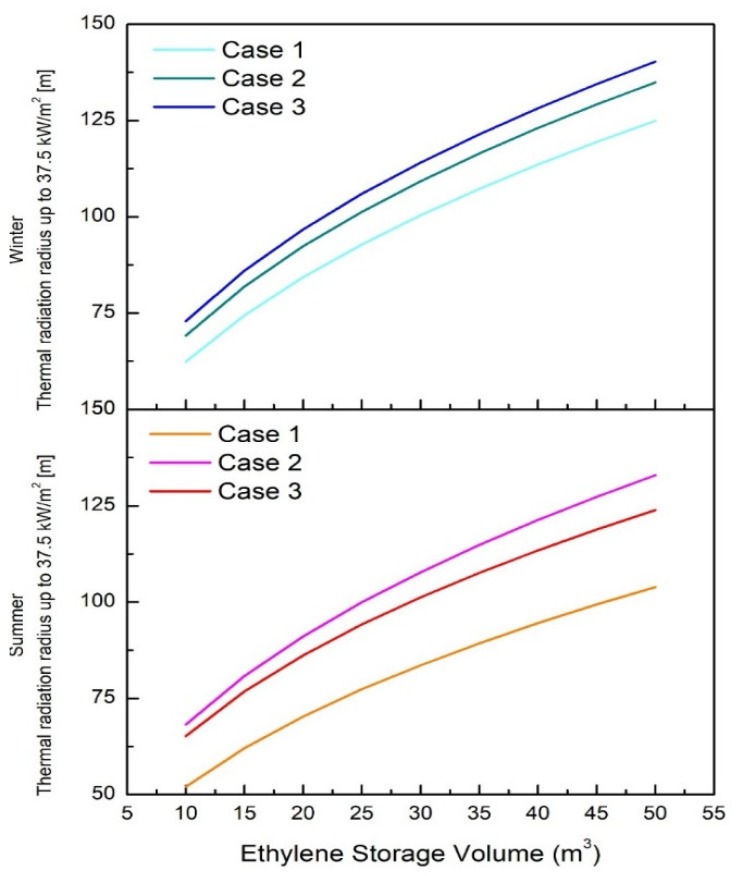
Thermal radiation radius up to 37.5 kW/m^2^ according to ethylene storage capacity in summer (40 °C) and winter (0 °C) conditions.

The simulation results for summer (40 °C) and winter (0 °C) are shown in Table 7, Figure 3, and the regression analysis with polynomial fitting is shown in Table 8.

For each case, the regression equation is expressed as Equations (4)–(9). Each calculation provided high interpretation results.

(4) $$\mathrm{Summer}\;\mathrm{Case}\;1\text{: }Y=32.91524+2.1227X-0.01428X^{2}$$

(5) $$\mathrm{Summer}\;\mathrm{Case}\;2\text{: }Y=44.33229+2.6596X-0.018X^{2}$$

(6) $$\mathrm{Summer}\;\mathrm{Case}\;3\text{: }Y=43.45616+2.43186X-0.01672X^{2}$$

(7) $$\mathrm{Winter}\;\mathrm{Case}\;1\text{: }Y=39.44695+2.54553X-0.01697X^{2}$$

(8) $$\mathrm{Winter}\;\mathrm{Case}\;2\text{: }Y=44.90677+2.69489X-0.01817X^{2}$$

(9) $$\mathrm{Winter}\;\mathrm{Case}\;3\text{: }Y=47.98425+2.77211X-0.01879X^{2}$$

**Figure 4:**
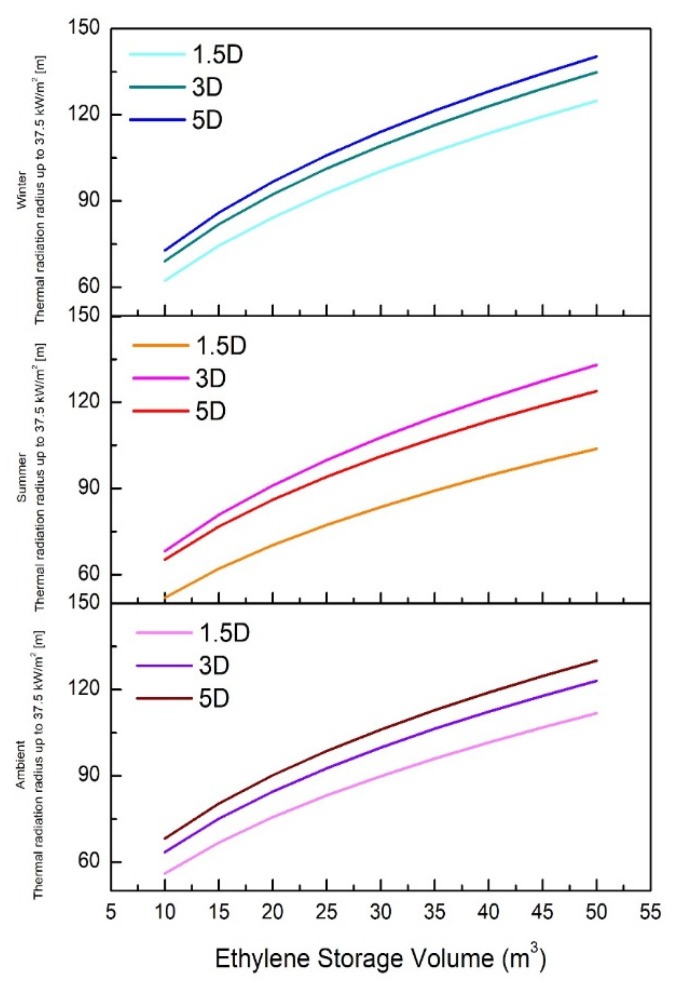
Thermal radiation radius up to 37.5 kW/m^2^ according to ethylene storage capacity in ambient temperature (25 °C), summer (40 °C), and winter (0 °C) conditions.

In the summer (40 °C) condition, a wind speed of 1.5 m/s showed a thermal radiation of 37.5 kW/m^2^, which is very different from other temperature conditions. Wind speeds of 5 and 3 m/s did not show large differences, but the thermal radiation was the largest at 3 m/s. However, in winter (0 °C) and ambient temperature (25 °C) conditions, the thermal radiation was confirmed to widen to 37.5 kW/m^2^ with increasing wind speed. Figure 4 presents the thermal radiation up to 37.5 kW/m^2^ for each wind speed case at ambient temperature (25 °C), summer (40 °C), and winter (0 °C) conditions.

PIP PNE00003, one of the most widely used engineering guidelines that does not consider weather conditions and ethylene storage capacity, suggests a safety distance of 46 m between the ethylene storage tank and process facility, which is underestimated irrespective of the storage and weather conditions. A safety distance of 106.75 m suggested by GAP.2.5.2 is valid only under certain capacity and weather conditions. Table 9 presents the analysis results for each weather condition through the polynomial fitting derived from this study.

## 4. Discussion

The separation distance in chemical plants is considered as inherent safety to prevent chain explosion [22]. In chemical plants, the storage tank handles a large amount of chemicals, so it should be considered first when designing the separation distance [23]. Since the separation distance is applied as a concept to prevent chain explosion, it is independent of toxic dispersion.

The design of chemical plant is divided into code-based design and performance-based design according to the approach. Following the consistent regulation along the global engineering guideline is called code-based design, and applying the actual performance considering various factors is called performance-based design [24]. Currently, the separation distance is dependent on the global engineering guidelines like other design fields of chemical plants. However, it is difficult to consider the global engineering guidelines as guaranteeing safety because no detailed factors are considered [25]. QRA is performed to complement the limitations of the global engineering guidelines [26,27]. PIP PNE00003 and GAP.2.5.2 are the global engineering guidelines in the separation distance field [6]. The storage tank capacity and atmospheric conditions are not considered in the separation distance between the storage tank and the process equipment presented in these guidelines. These guidelines just categorize only by item type, such as storage tank or pump and so on, and provide only consistent separation distance between categories. Therefore, this study used QRA to analyze the separation distance between storage tank and process equipment.

The influence range was analyzed for thermal radiation up to 37.5 kW/m^2^ in the case of a fire due to contact with an ignition source. At ambient temperature, atmospheric stability did not affect the radius of thermal radiation, and the same results were obtained for summer and winter conditions. The atmospheric stability was simulated by changing only the wind speed with fixed D.

Polynomial fitting of the radius of thermal radiation for each weather case, according to the capacities of ethylene storage under ambient temperature, summer, and winter conditions, provided results with an average reliability of 99.892%. In the summer, changes in the radius of thermal radiation according to wind speed were not observed, but in the case of ambient temperature and winter, the radius of thermal radiation increased with increase in wind speed.

In comparison with existing international engineering guidelines, a safety distance of 46 m (PIP PNE00003) is underestimated in all cases of capacities and atmospheric conditions, and a distance of 106.75 m (GAP.2.5.2) does not guarantee safety when the capacities presented in Table 9 are exceeded and the weather conditions are not met.

Therefore, when setting the distance between the ethylene storage tank and the process equipment in the future, it is possible to meet the safety distance that cannot be established by existing engineering practices using the result of the Phast simulation, considering weather conditions. This study limited only one substance to be stored (ethylene), climatic conditions to three categories (ambient temperature, summer, winter), and wind speed conditions to three categories (1.5, 3, 5 m/s). However, it is expected that the same approach to the storage of different materials and different atmospheric conditions through the methodology of this study would be able to supplement the limitations of the global engineering guidelines.

## 5. Conclusions

Ethylene is the base material of the chemical industry and can be obtained through the process of naphtha cracking or ethylene cracking. In addition, ethylene is one of the basic constituents of a chemical plant as the scale of ethylene production is considered a measure of the chemical-industry development of a country. Ethylene occurs in the gaseous phase at ambient temperature but may be converted to the liquid phase under conditions of high pressure and low temperature, which is convenient for storage and transportation. A large amount of ethylene is stored in ethylene storage tanks, making them a major item to be considered when determining the safety distance.

The separation distance according to thermal radiation was applied in the previous studies. For thermal radiation, the explosive characteristic of the material is the most important parameter. The main factors that determine the explosiveness of each material are the Lower Flammable Limit (LFL) and the molecular weight for the dispersion radius. The environmental factors may include toxic dispersion, but this was not considered in this study because it indicated that it limited thermal radiation.

Global guidelines suggest that 37.5 kW/m^2^ is the limit of thermal radiation that the process equipment can experience. When leakage occurs in the ethylene storage tank, a pool forms, and fire or explosion may occur on contact with an ignition source. This study investigated the safety distance between ethylene storage tanks and process equipment. The radius of thermal radiation (37.5 kw/m^2^) was simulated using Phast. The trends were also analyzed for different ethylene storage capacities and weather conditions.

In the results, atmospheric stability did not affect the radius of thermal radiation. Under ambient temperature, the radius of thermal radiation increased with increase in wind speed in winter but not in summer. In addition, a regression equation was derived through polynomial fitting for each case, and all regression-equation calculations exhibited a high interpretation reliability of 99.892% on average.

In the previous studies, there has been research of the separation distance by thermal radiation. However, there have been no studies comparing the separation distance suggested by the global engineering guidelines. In the process safety engineering field like separation distance, the academic part is important, but the practical part must also be considered. This is because if only the academic part is considered, the possibility of applicability in the work-site operation is likely to be excluded. This study introduced the contents of global engineering guidelines used in work-site operation and introduced methods to compensate for the limitations of these guidelines.

International engineering guidelines include PIP PNE00003 and GAP.2.5.2. The safety distance between the ethylene storage tank and process equipment presented in PIP PNE00003 is underestimated, irrespective of the ethylene storage capacity and atmospheric conditions. The safety distance suggested by GAP.2.5.2 is applicable only under certain weather conditions and storage capacity.

Fundamentally, both PIP PNE00003 and GAP.2.5.2 offer consistent separation distances that do not consider ethylene storage capacity or weather conditions. Therefore, it is necessary to include a simulation tool, such as QRA, for precise safety-distance determination.

## Figures and Tables

**Table 1:** Thermal radiation rating adversely affecting chemical plant equipment.

Source	Description
Assessment Guidance: Dinenno (1982)	37.5 kW/m^2^: Equipment damage (Gelderblom, 1980)
Effects of Thermal Radiation—World Bank: 1985	37.5 kW/m^2^: Sufficient to cause damage to process equipment
Design and Assessment Guidance—BS 5980: 1990	37.5 kW/m^2^: Intensity at which damage is caused to process equipment

**Table 2:** Process condition of ethylene storage tank.

Operating Condition	Variable
Operating temperature	−103.2 °C
Operating pressure	0.055 kgf/cm^2^g
Storage capacity	10, 15, 20, 25, 30, 35, 40, 45, 50 m^3^

**Table 3:** Wind speed and atmospheric stability applied to the simulation.

Weather Case	Wind Speed (m/s)	Atmospheric Stability
Case 1	1.5	D
Case 2		F
Case 3	3	D
Case 4		F
Case 5	5	D
Case 6		F

**Table 4:** Simulation results at ambient temperature (25 °C).

Weather Case	Ethylene Storage Volume (m^3^)	Distance Downwind to Intensity Level 3 (37.5 kW/m^2^) (m)
Case 1 (1.5/D) and Case 2 (1.5/F)	10	55.9493
	15	66.7322
	20	75.5822
	25	83.1903
	30	89.9265
	35	96.0267
	40	101.657
	45	106.838
	50	111.732
Case 3 (3/D) and Case 4 (3/F)	10	63.4705
	15	75.0616
	20	84.5516
	25	92.666
	30	99.8702
	35	106.335
	40	112.298
	45	117.83
	50	123.013
Case 5 (5/D) and Case 6 (5/F)	10	68.2042
	15	80.3337
	20	90.2283
	25	98.6466
	30	106.096
	35	112.864
	40	119.004
	45	124.654
	50	130.074

**Table 5:** Regression analysis results according to polynomial fitting: Simulation under ambient temperature (25 °C) condition.

Weather Case	Intercept		B1		B2		Statistics
	Value	Standard Error	Value	Standard Error	Value	Standard Error	Adj. R-Square
Case 1 and 2	35.46308	1.1432	2.2785	0.08498	−0.01528	0.00139	0.99894
Case 3 and 4	41.49652	1.26058	2.44807	0.09371	−0.0166	0.00153	0.99887
Case 5 and 6	45.27472	1.3456	2.55867	0.10003	−0.01752	0.00164	0.99881

**Table 6:** Wind speed and atmospheric stability applied to simulations in summer (40 °C) and winter (0 °C) conditions.

Weather Case	Wind Speed (m/s)	Temperature (°C)
Summer Case 1	1.5	40
Summer Case 2	3	
Summer Case 3	5	
Winter Case 1	1.5	0
Winter Case 2	3	
Winter Case 3	5	
Ambient Case 1	1.5	25
Ambient Case 2	3	
Ambient Case 3	5	

**Table 7:** Simulation results in summer (40 °C) and winter (0 °C) conditions.

Weather Case	Ethylene Storage Volume (m^3^)	Distance Downwind to Intensity Level 3 (37.5 kW/m^2^) (m)
Summer Case 1	10	51.9886
	15	62.0542
	20	70.2753
	25	77.3549
	30	83.593
	35	89.2994
	40	94.5108
	45	99.3632
	50	103.851
Summer Case 2	10	68.2257
	15	80.8162
	20	91.0897
	25	99.9327
	30	107.739
	35	114.842
	40	121.359
	45	127.348
	50	132.939
Summer Case 3	10	65.2316
	15	76.791
	20	86.1365
	25	94.1513
	30	101.218
	35	107.59
	40	113.409
	45	118.836
	50	123.868
Winter Case 1	10	62.3411
	15	74.4295
	20	84.2913
	25	92.8115
	30	100.386
	35	107.262
	40	113.569
	45	119.4
	50	124.91
Winter Case 2	10	69.1151
	15	81.8961
	20	92.3217
	25	101.286
	30	109.233
	35	116.433
	40	123.039
	45	129.183
	50	134.869
Winter Case 3	10	72.8759
	15	86.0084
	20	96.7068
	25	105.938
	30	114.078
	35	121.426
	40	128.206
	45	134.439
	50	140.301

**Table 8:** Regression analysis results according to polynomial fitting: Simulation under summer (40 °C) and winter (0 °C) conditions.

Weather Case	Intercept		B1		B2		Statistics
	Value	Standard Error	Value	Standard Error	Value	Standard Error	Adj. R-Square
Summer Case 1	32.91524	1.06629	2.1227	0.07927	−0.01428	0.0013	0.99894
Summer Case 2	44.33229	1.32374	2.6596	0.0984	−0.018	0.00161	0.99895
Summer Case 3	43.45616	1.28213	2.43186	0.09531	−0.01672	0.00156	0.9988
Winter Case 1	39.44695	1.26765	2.54553	0.09423	−0.01697	0.00154	0.99897
Winter Case 2	44.90677	1.35602	2.69489	0.1008	−0.01817	0.00165	0.99893
Winter Case 3	47.98425	1.40369	2.77211	0.10435	−0.01879	0.00171	0.99891

**Table 9:** Storage capacity of ethylene to satisfy the safety distance of GAP.2.5.2 according to atmospheric conditions.

Weather Condition		Ethylene Capacity Where the Safety Distance of GAP.2.5.2 Can Be Conservative (m^3^)
Ambient	25 °C and 1.5D	44.6 or less
	25 °C and 3D	34.9 or less
	25 °C and 5D	30.3 or less
Summer	40 °C and 1.5D	55.5 or less
	40 °C and 3D	29.2 or less
	40 °C and 5D	33.9 or less
Winter	0 °C and 1.5D	34.2 or less
	0 °C and 3D	28.3 or less
	0 °C and 5D	25.6 or less
